# Supplementary material for: Hepatocyte growth factor derived from senescent cells attenuates cell competition-induced apical elimination of oncogenic cells
Source: Nat Commun. 2022 Jul 18;13:4157. doi: 10.1038/s41467-022-31642-4 (PMC9293948; doi:10.1038/s41467-022-31642-4)
Supplement: Supplementary file 1 — Supplementary Information [file 41467_2022_31642_MOESM1_ESM.pdf]

Supplementary Materials for

Hepatocyte growth factor derived from senescent cells attenuates cell competition-induced apical elimination of oncogenic cells

Nanase Igarashi, Kenichi Miyata, Tze Mun Loo, Masatomo Chiba, Aki Hanyu, Mika Nishio, Hiroko Kawasaki, Hao Zheng, Shinya Toyokuni, Shunsuke Kon, Keiji Moriyama, Yasuyuki Fujita, ***Akiko Takahashi

This supplementary file includes:

Supplementary Figure S1 to S11

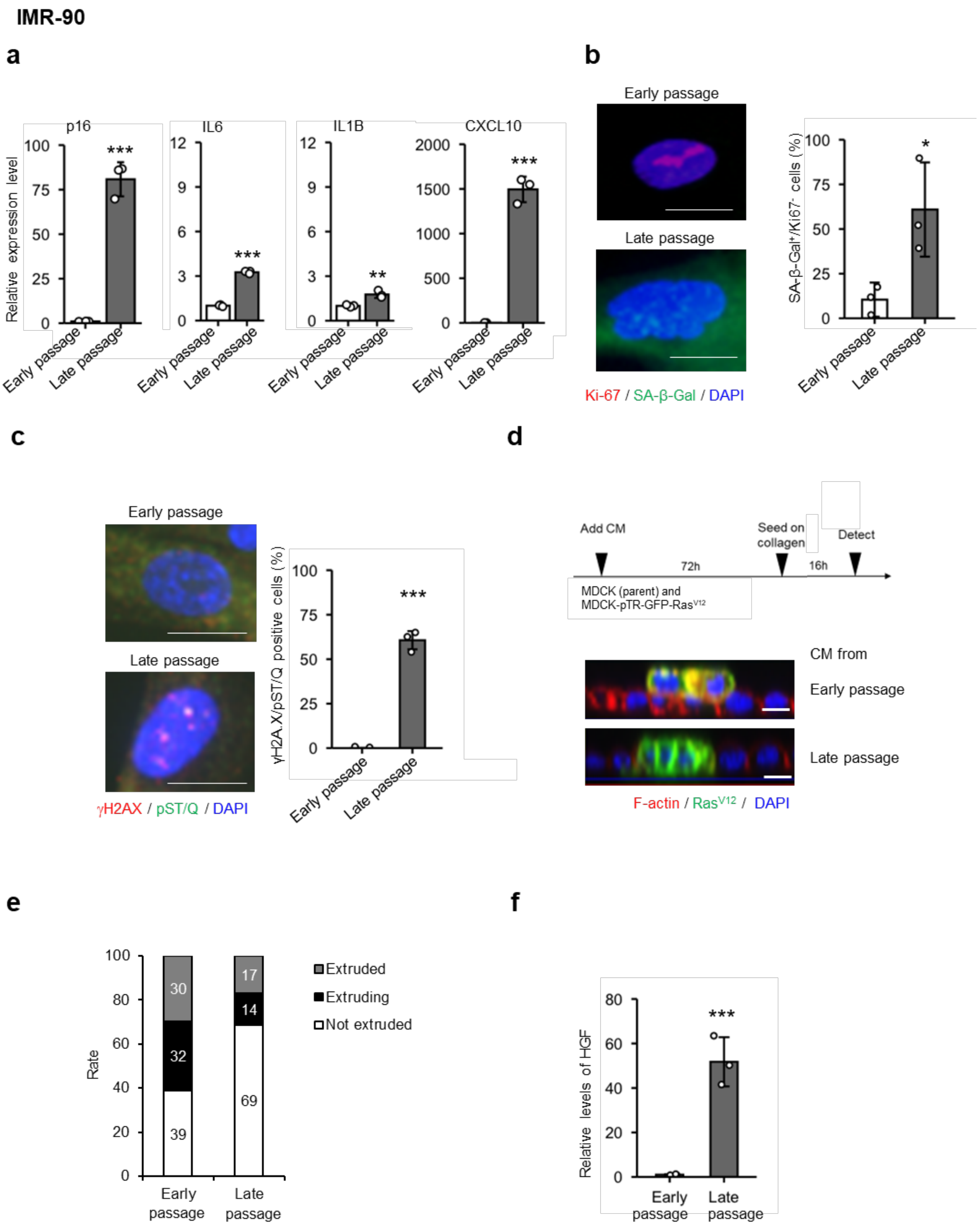

Figure S1. SASP factors derived from senescent IMR-90 cells prevent apical extrusion of Ras^{V12} cells

a–c, Pre-senescent IMR-90 cells were rendered senescent by passaging (late passage). These cells were then subjected to RT-qPCR analysis of p16 and SASP factor gene expression [p16, $p <$

0.001; IL6, $p < 0.001$; IL1B, $p < 0.01$; CXCL10, $p < 0.001$] (**a**) or immunofluorescence staining for markers of senescent cell cycle arrest [Ki67 (red), SA-β-gal (green) and DAPI (blue)], $p = 0.0036$ (**b**) and DNA damage [γ-H2AX (red), phosphor-Ser/Thr ATM/ATR (pST/Q) substrate (green) and DAPI (blue)], $p < 0.001$ (**c**). Representative data from three independent experiments are shown. The histograms indicate the percentage of Ki67-negative and SA-β-gal-positive cells (**b**) and nuclei containing more than three foci positive for γ-H2AX and pST/Q staining (**c**). At least 100 cells were scored per group (**b, c**). Error bars indicate the mean ± standard deviation of three independent measurements (**a–c**). Scale bar, 10 μm. **d**, **e**, MDCK (parent) and MDCK-pTR GFP-Ras^{V12} cells were separately treated with CM derived from early or late passage senescent IMR-90 cells for 3 days. The treated MDCK (parent) and MDCK-pTR GFP-Ras^{V12} cells were mixed at a ratio of 50:1 and cultured on type-I collagen gels. These MDCK cells were stained with phalloidin (F-actin, red) and DAPI (blue) after 16 h of incubation with tetracycline to induce Ras^{V12} in MDCK-pTR GFP-Ras^{V12} cells (**d**). Quantification of the apically extruded, extruding, or not extruded MDCK-pTR GFP-Ras^{V12} cells from a monolayer of MDCK normal cells was performed (**e**). **f**, ELISA was used to analyze HGF production in early or late passage senescent IMR-90 cells. $p = 0.0014$. *$P < 0.05$, **$P < 0.01$, ***$P < 0.001$ by the unpaired two-sided *t*-test.

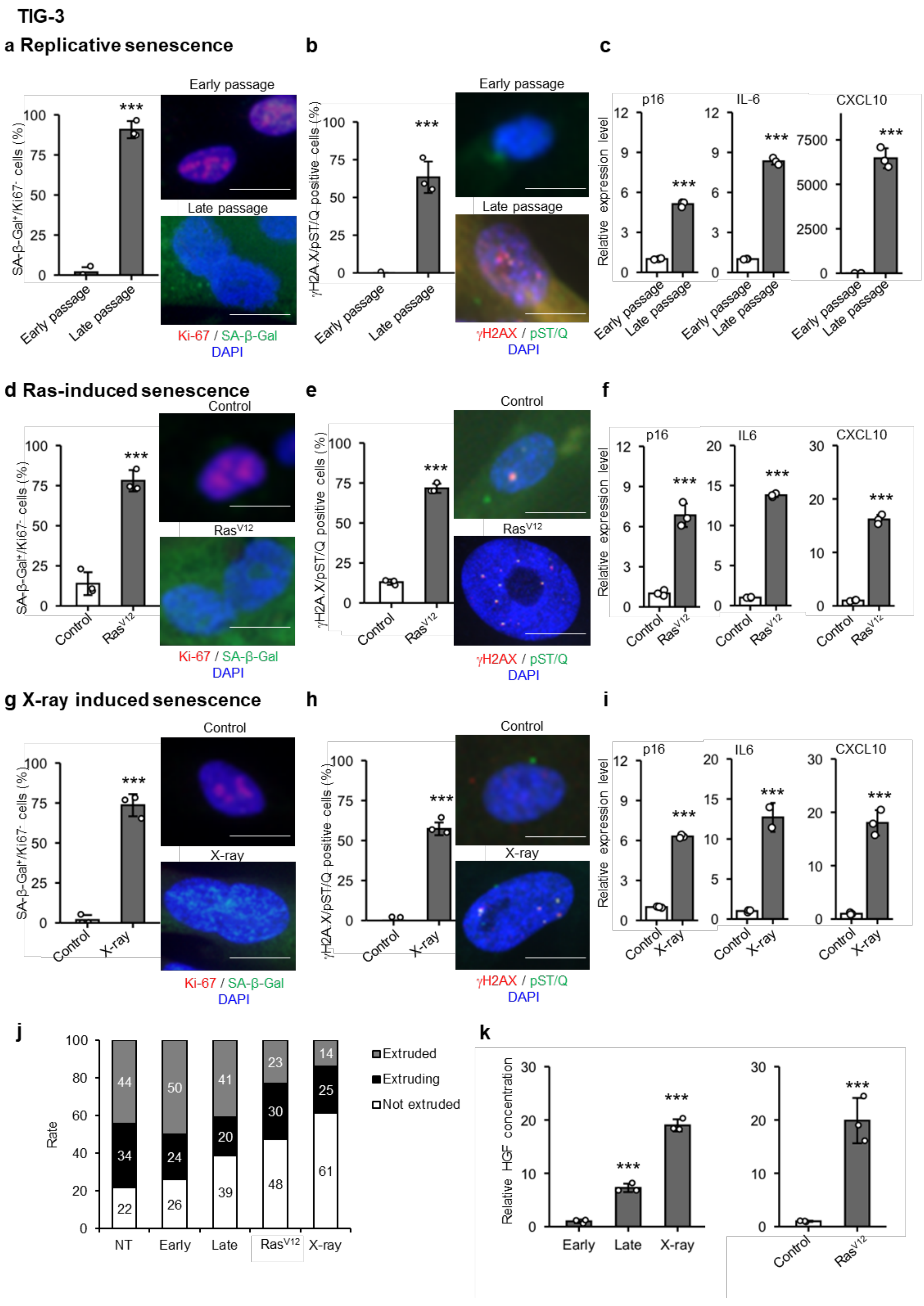
TIG-3
a Replicative senescence
b
c
SA-β-Gal+/Ki67- cells (%)
Early passage
Late passage
Ki-67 / SA-β-Gal
DAPI
γH2A.X/pST/Q positive cells (%)
γH2AX / pST/Q
Relative expression level
p16
IL-6
CXCL10
d Ras-induced senescence
e
f
Control
RasV12
IL6
g X-ray induced senescence
h
i
X-ray
j
Rate
Extruded
Extruding
Not extruded
NT
Early
Late
k
Relative HGF concentration

Figure S2. SASP factors derived from senescent TIG-3 cells inhibit apical extrusion of Ras^{V12} cells

a–i, Pre-senescent TIG-3 cells were rendered senescent by either serial passage (late passage) (**a–c**) [$p < 0.001$ (**a**); $p < 0.001$ (**b**); p16, $p < 0.001$ (**c**) ; IL6, $p < 0.001$ (**c**) ; CXCL10, $p < 0.001$ (**c**)], ectopic expression of oncogenic Ras (+HRasV12) (**d–f**) [$p < 0.001$ (**d**); $p < 0.001$ (**e**); p16, $p < 0.001$ (**f**) ; IL6, $p < 0.001$ (**f**) ; CXCL10, $p < 0.001$ (**f**)] or X-ray irradiation (10 Gy) (**g–i**) [$p < 0.001$ (**g**); $p < 0.001$ (**h**); p16, $p < 0.001$ (**i**) ; IL6, $p < 0.001$ (**i**) ; CXCL10, $p < 0.001$ (**i**)]. These cells were then subjected to immunofluorescence staining for markers of senescent cell cycle arrest [Ki67 (red), SA-β-gal (green) and DAPI (blue)] (**a**, **d** and **g**) and DNA damage [γ-H2AX (red), phosphor-Ser/Thr ATM/ATR (pST/Q) substrate (green) and DAPI (blue)] (**b**, **e** and **h**) or to RT-qPCR analysis of p16 and SASP factor gene expression (**c**, **f** and **i**). Representative data from three independent experiments are shown. The histograms indicate the percentage of Ki67-negative and SA-β-gal-positive cells (**a**, **d**, and **g**) and nuclei containing more than three foci positive for γ-H2AX and pST/Q staining (**b**, **e**, and **h**). At least 100 cells were scored per group. Error bars indicate the mean ± standard deviation of three independent measurements (**a–i**). Scale bar, 10 μm. **j**, MDCK (parent) and MDCK-pTR GFP-Ras^{V12} cells were separately treated with CM derived from early passage, late passage, Ras-induced senescent or X-ray induced senescent TIG-3 cells for three days. The treated MDCK (parent) and MDCK-pTR GFP-Ras^{V12} cells were mixed at a ratio of 50:1 and cultured on type-I collagen gels. These MDCK cells were stained with phalloidin and DAPI after 16 h of incubation with tetracycline to induce Ras^{V12} in MDCK-pTR GFP-Ras^{V12} cells. Quantification of the apically extruded, extruding, or not extruded MDCK-pTR GFP-Ras^{V12} cells from a monolayer of MDCK normal cells was performed. **k**, ELISA was performed to analyze HGF production in early passage, late passage, X-ray induced senescent, or Ras^{V12}-induced senescent TIG-3 cells. $p < 0.001$ (Late), $p < 0.001$ (X-ray), $p < 0.001$ ($HRas^{V12}$). ***$P < 0.001$ by the unpaired two-sided *t*-test.

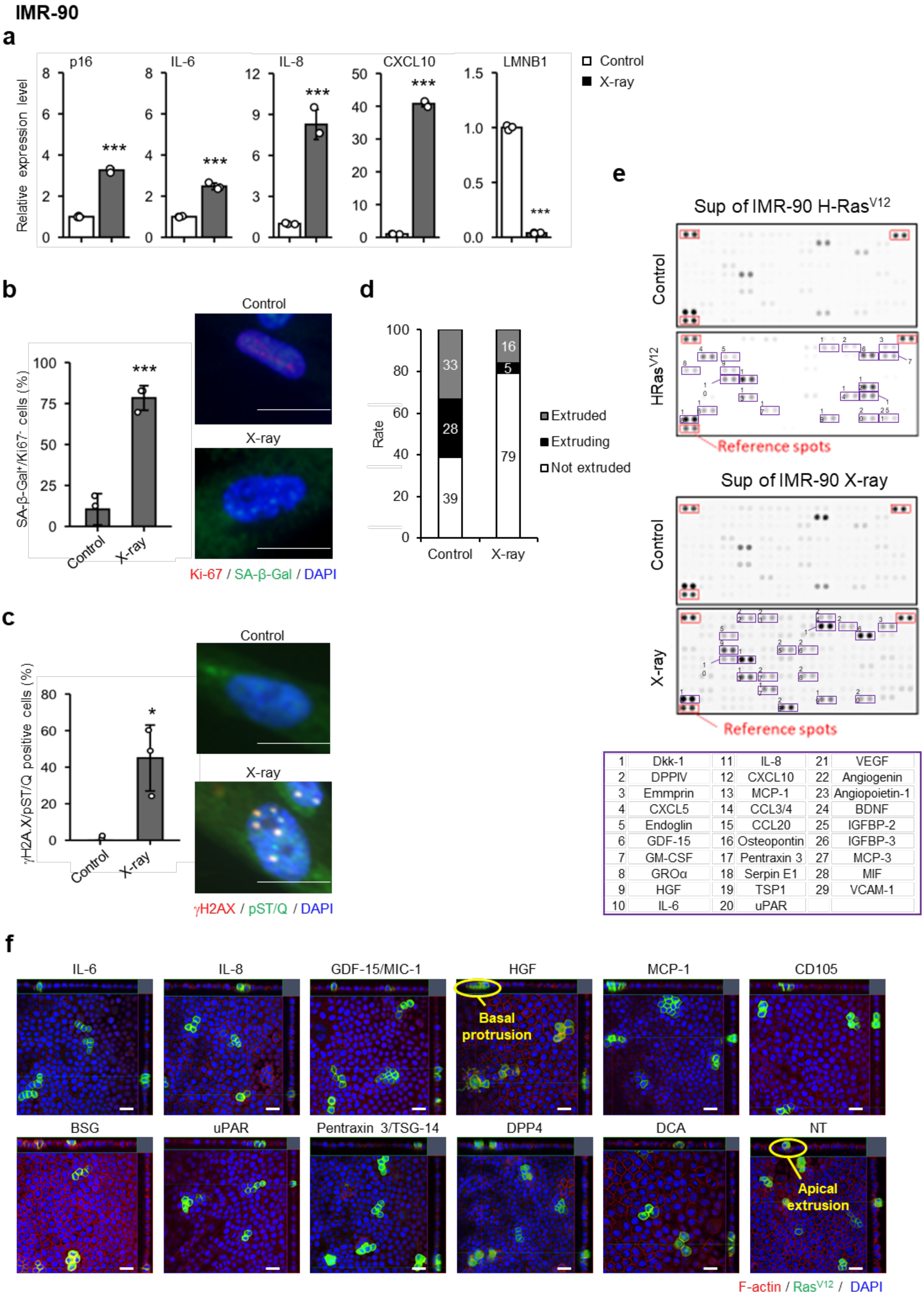

1	Dkk-1	11	IL-8	21	VEGF
2	DPPIV	12	CXCL10	22	Angiogenin
3	Emmprin	13	MCP-1	23	Angiopoietin-1
4	CXCL5	14	CCL3/4	24	BDNF
5	Endoglin	15	CCL20	25	IGFBP-2
6	GDF-15	16	Osteopontin	26	IGFBP-3
7	GM-CSF	17	Pentraxin 3	27	MCP-3
8	GROα	18	Serpin E1	28	MIF
9	HGF	19	TSP1	29	VCAM-1
10	IL-6	20	uPAR		

Figure S3. HGF is responsible for inhibiting apical extrusion of Ras^{V12} cells

a–c, Presenescent IMR-90 cells were rendered senescent by 10-Gy X-ray irradiation. After 10 days, these cells were then subjected to RT-qPCR analysis of $p16^{INK4a}$, SASP factor gene and LMB1 expression (**a**) [p16, $p < 0.001$; IL-6, $p < 0.001$; IL-8, $p < 0.001$; CXCL10, $p < 0.001$; LMNB1, $p < 0.001$], immunofluorescence staining for markers of senescent cell cycle arrest [Ki67 (red), SA-β-gal (green) and DAPI (blue)] (**b**) and DNA damage [γ-H2AX (red), phosphor-Ser/Thr ATM/ATR (pST/Q) substrate (green) and DAPI (blue)]. Representative data from three independent experiments are shown. The histograms indicate the percentage of Ki67-negative and SA-β-gal-positive cells, $p < 0.001$. (**b**) and nuclei containing more than three foci positive for γ-H2AX and pST/Q staining, $p = 0.012$. (**c**). At least 100 cells were scored per group (**b** and **c**). Error bars indicate the mean ± standard deviation of three independent measurements (**a–c**). Scale bar, 10 μm (**b** and **c**). *$P < 0.05$, ***$P < 0.001$ by the unpaired two-sided *t*-test. **d**, MDCK (parent) and MDCK-pTR GFP-Ras^{V12} cells were separately treated with CM derived from X-ray induced senescent IMR-90 cells for 3 days. The treated MDCK (parent) and MDCK-pTR GFP-Ras^{V12} cells were mixed at a ratio of 50:1 and cultured on type-I collagen gels. These MDCK cells were stained with phalloidin and DAPI after 16 h of incubation with tetracycline to induce Ras^{V12} in MDCK-pTR GFP-Ras^{V12} cells. Quantification of the apically extruded, extruding, or not extruded MDCK-pTR GFP-Ras^{V12} cells from a monolayer of MDCK normal cells. **e**, Cytokine array analysis of CM derived from oncogene- (upper) and X-ray induced (lower) senescent IMR-90 cells (n=1). Human cytokine array analysis of multiple cytokines secreted by MDCK-pTR GFP-Ras^{V12} cells after treatment with CM derived from senescent IMR-90 cells. The image is a human cytokine array coordinate displaying the location of detected cytokines. **f**, MDCK and MDCK-pTR GFP-Ras^{V12} cells were treated with 250 ng/ml of each recombinant protein for 3 days. The treated MDCK and MDCK-pTR GFP-Ras^{V12} cells were mixed at a ratio of 50:1 and cultured on type-I collagen gels. These MDCK cells were stained with phalloidin (F-actin, red) and DAPI (blue) after 16 h of incubation with tetracycline to induce Ras^{V12} in MDCK-pTR GFP-Ras^{V12} cells. Scale bar, 20 μm.

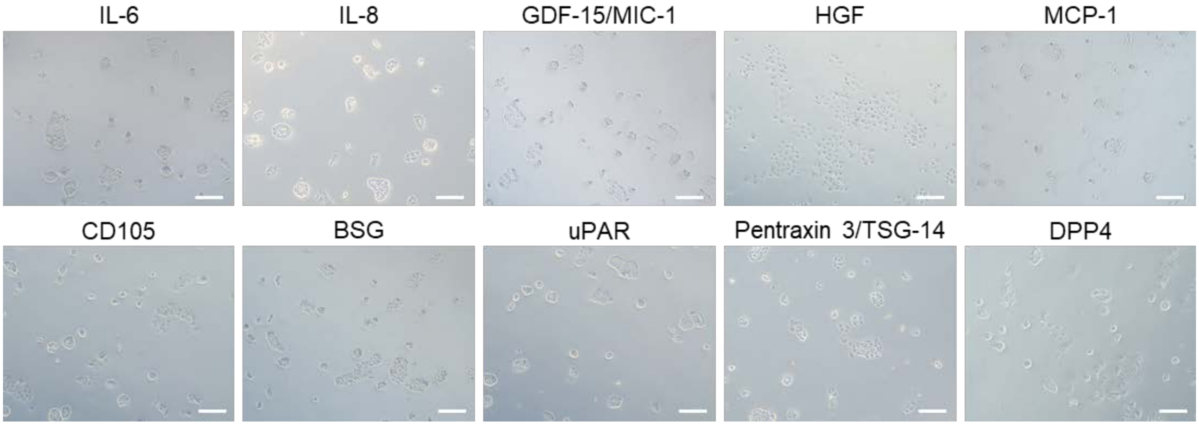

Figure S4. HGF induces EMT-like morphological change in Ras^{V12} cells
The appearance of MDCK-pTR GFP-Ras^{V12} cells treated with 250 ng/ml of each recombinant protein was indicated for 24 h. Scale bar, 200 μm.

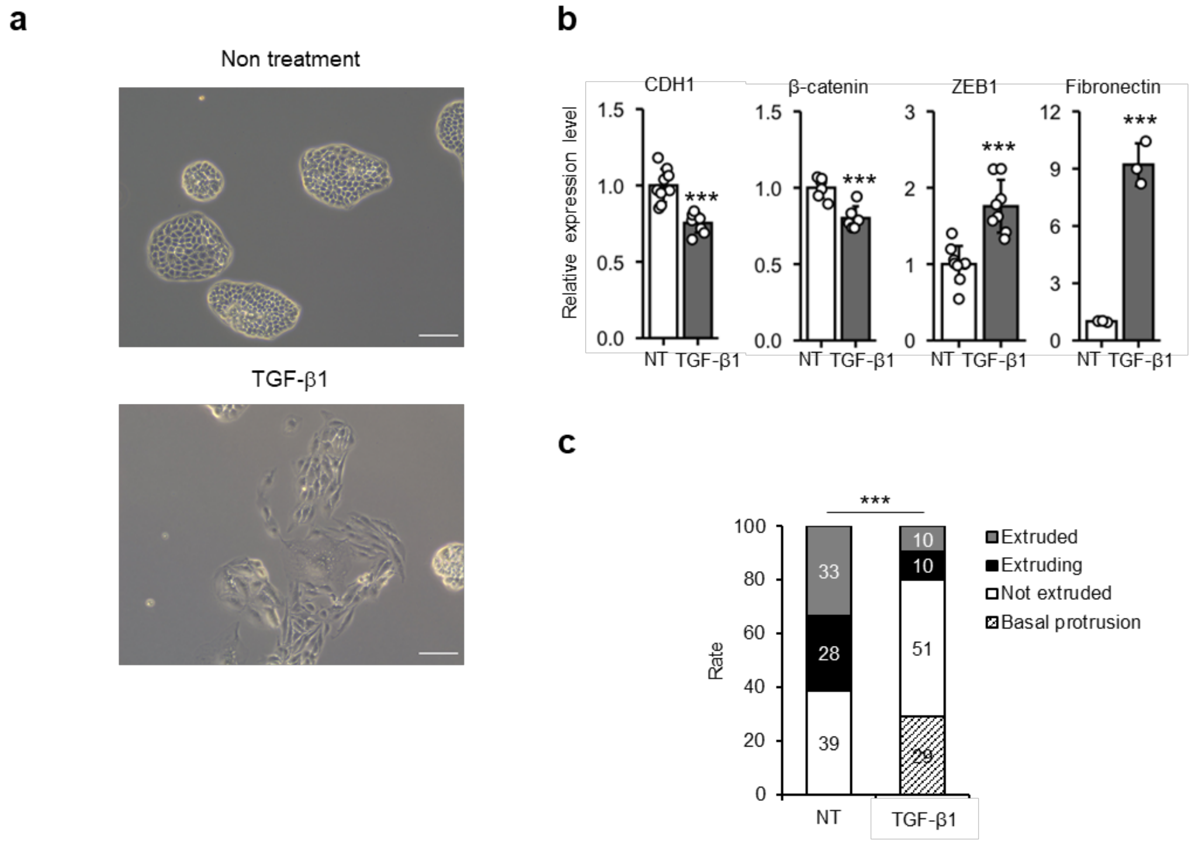

Figure S5. TGF-β1 inhibits apical extrusion of Ras^{V12} cells

a, The appearance of MDCK-pTR GFP-Ras^{V12} cells with or without 10 ng/ml of TGF-β1 for 2 days. Scale bar; 100 μm. **b**, RT-qPCR analysis of EMT markers in treated MDCK-pTR GFP-Ras^{V12} cells with 10 ng/mL TGF-β1 for 2 days. Representative data from three independent experiments are shown. Error bars indicate the mean ± standard deviation. CDH1, $p < 0.001$; β-catenin, $p < 0.001$; ZEB1, $p < 0.001$, Fibronectin, $p < 0.001$. **c**, These MDCK-pTR GFP-Ras^{V12} cells were mixed at a ratio of 50:1 and cultured on type-I collagen gels and stained with phalloidin and DAPI after 16 h of incubation with tetracycline to induce Ras^{V12}. Quantification of the apically extruded, extruding, not extruded, or basally protruding MDCK-pTR GFP-Ras^{V12} cells from a monolayer of normal MDCK cells (**c**). $p < 0.001$. *$P < 0.05$, **$P < 0.01$, or ***$P < 0.001$ by the unpaired two-sided *t*-test (**b**) and chi-square test (**c**).

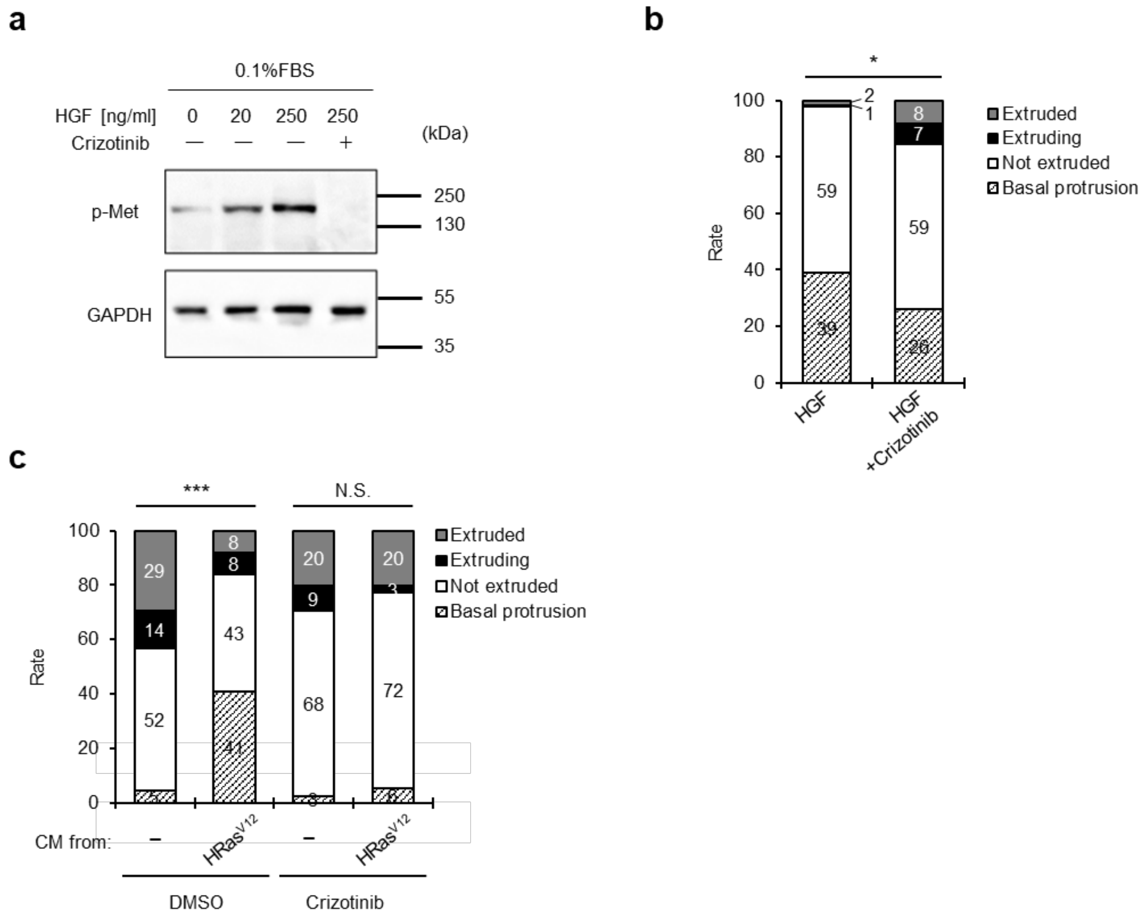

Figure S6. Crizotinib attenuates the effect of the senescent secretome in cell competition
a, **b**, MDCK-pTR GFP-Ras^{V12} cells were treated with 20 or 250 ng/ml of HGF or CM derived from oncogene-induced senescent IMR-90 cells for 3 days. Dimethyl sulfoxide or 100 nM of crizotinib was added before the cell competition assay. These treated MDCK (parent) and MDCK-pTR GFP-Ras^{V12} cells were mixed at a ratio of 50:1 and cultured on type-I collagen gels. These MDCK cells were stained with phalloidin and DAPI after 16 h of incubation with tetracycline to induce Ras^{V12} in MDCK-pTR GFP-Ras^{V12} cells. These cells were subjected to western blotting using the antibodies shown toward the left (**a**). Quantification of the apically extruded, extruding, not extruded, or basally protruding MDCK-pTR GFP-Ras^{V12} cells from a monolayer of MDCK normal cells (**b**). $p = 0.013$. **c**, MDCK (parent), and MDCK-pTR GFP-Ras^{V12} cells were treated with CM derived from proliferating or oncogene-induced senescent IMR-90 cells with or without 100 nM crizotinib treatment. These cells were subjected to cell competition assay and quantification of the apically extruded, extruding, not extruded, or basally protruding MDCK-pTR GFP-Ras^{V12} cells from a monolayer of normal MDCK cells (**c**). $p < 0.001$ (DMSO); $p = 0.25$ (Crizotinib). *$P < 0.05$, ***$P < 0.001$, or not significant (N.S.) by chi-square test.

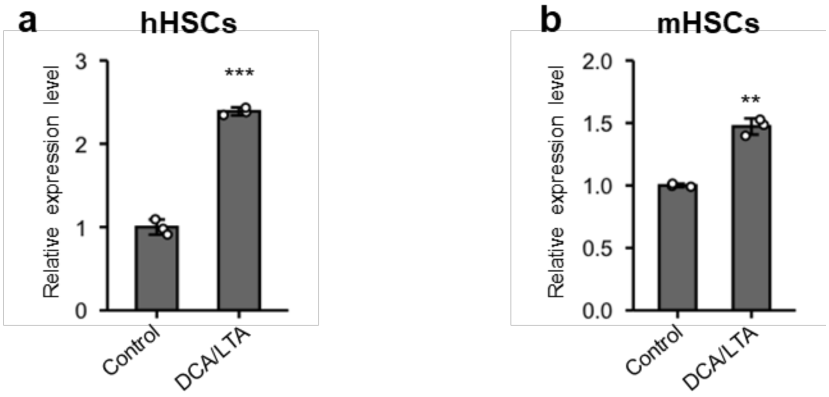

Figure S7. DCA/LTA treatment induces HGF expression in human and murine HSCs
Human (**a**) and murine (**b**) primary HSCs were incubated with deoxy cholic acid (DCA) for 6 days to induce cellular senescence and then lipoteichoic acid (LTA) for 6 hours before harvest. These cells were then subjected to qPCR analysis of HGF expression. Representative data from three independent experiments are shown. Error bars indicate the mean +standard deviation of three independent measurements for all graphs. $p < 0.001$ (hHSCs); $p < 0.001$ (mHSCs). **$P < 0.01$, ***$P < 0.001$ by the unpaired two-sided t-test.

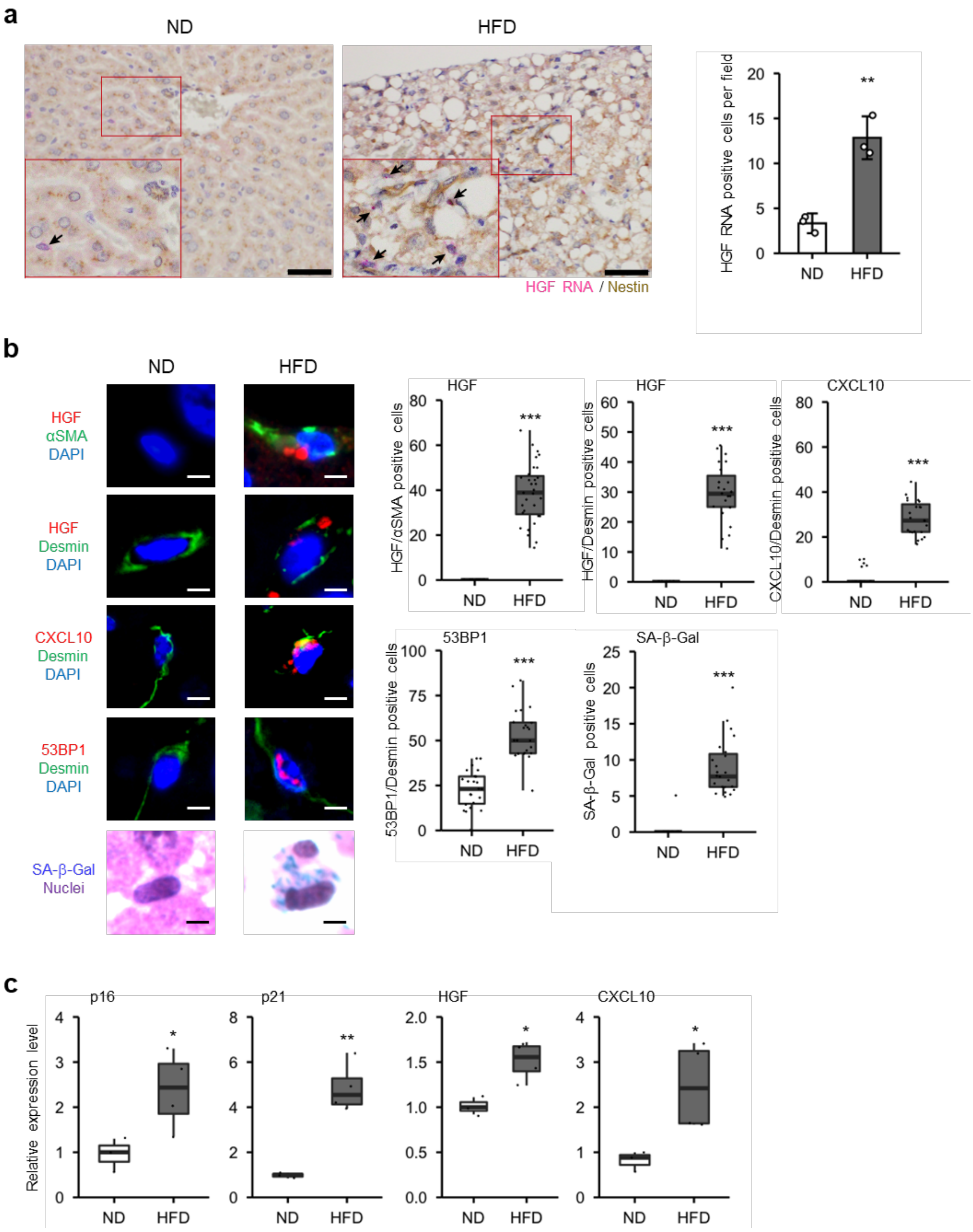

Figure S8. HFD induces cellular senescence and HGF expression in HSCs
C57BL/6 mice were fed ND or HFD for 3 months, euthanized, and their livers were subjected to RNA *in situ* hybridization (ISH) (**a**), immunofluorescence analysis (**b**), or RT-qPCR analysis (**c**). **a**, Representative and magnified (×100) images or quantified data of RNA-ISH with HGF RNA probe in liver specimens. Black arrows indicate HGF mRNA-positive cells. Scale bar, 50 μm. For each group (n=3), ≥9 images were randomly selected. HGF RNA-positive cells and selected areas were analyzed using NIH ImageJ. HGF RNA-positive cells per field were calculated as the number of total positive cells to the area fraction of the selected field. Bars indicate mean values and standard

error of the mean. Data were analyzed using the Student t-test, **$P < 0.01$. **b**, HSCs were visualized using α-SMA or desmin staining (green), and cell nuclei were stained using DAPI (blue). The histograms indicate percentages of α-SMA or desmin-expressing cells that were positive for the indicated markers. Scale bars, 5 μm. Data of three to four individual mice in each group are represented as mean ± standard deviation. HGF/α-SMA, n=31 (ND), n=31 (HFD) [$p < 0.001$]; HGF/Desmin, n=23 (ND), n=23 (HFD) [$p < 0.001$]; CXCL10/Desmin, n=23 (ND), n=23 (HFD) [$p < 0.001$]; 53BP1/Desmin, n=23 (ND), n=23 (HFD) [$p < 0.001$]; SA-β-Gal, n=23 (ND), n=23 (HFD) [$p < 0.001$]. More than 100 cells were counted in each field for statistical analysis. **c**, qPCR analysis of relative mRNA levels of p16, p21, HGF, and CXCL10 in ND or HFD-fed mouse livers (n=3). Data of three individual mice were shown as mean ±SD. $p = 0.047$ (p16); $p < 0.002$ (p21); $p < 0.014$ (HGF); $p < 0.037$ (CXCL10). *$P < 0.05$, **$P < 0.01$, or ***$P < 0.001$ by the unpaired two-sided *t*-test (**b**, **c**).

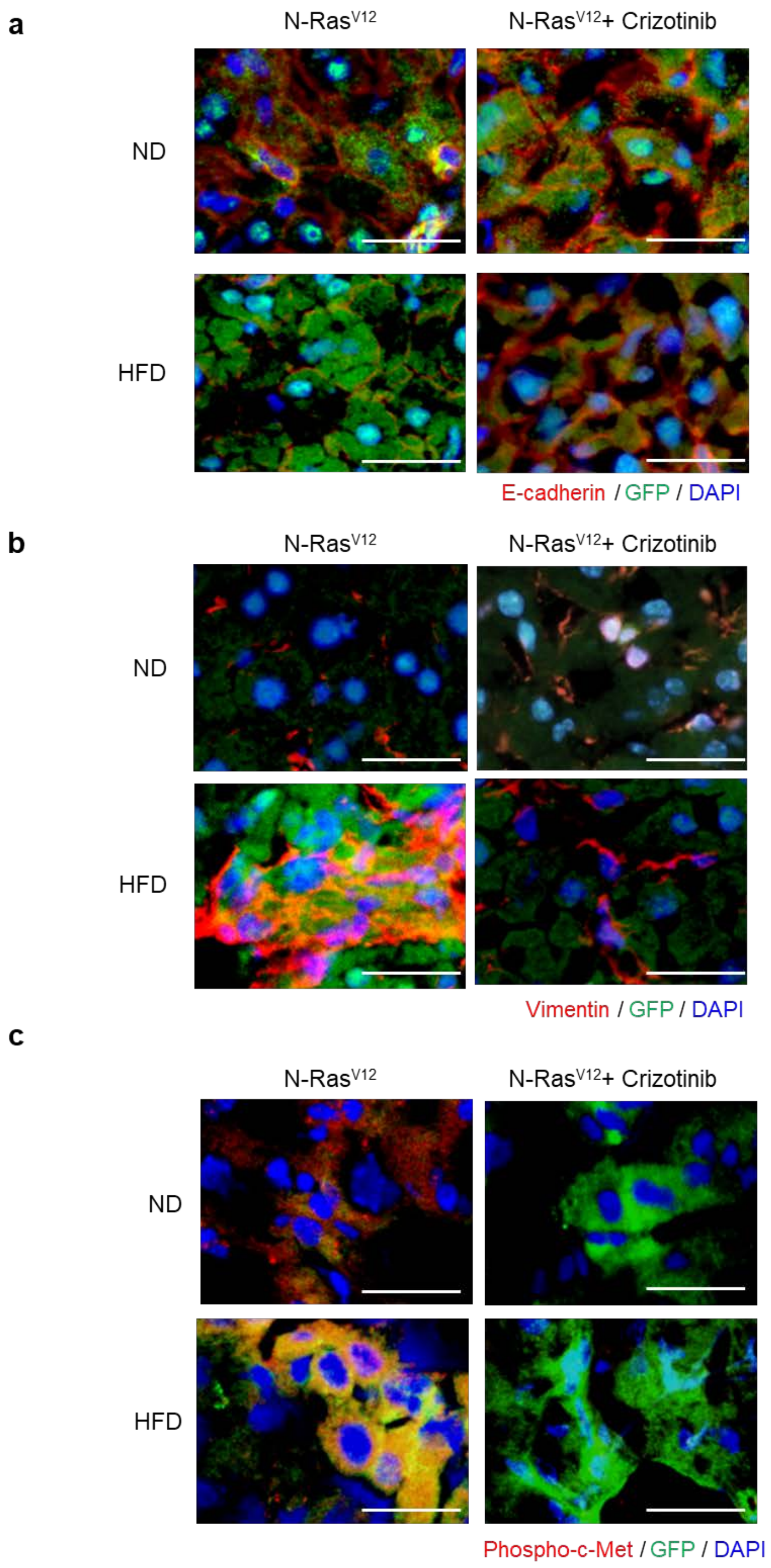

Figure S9. HFD induces EMT in Ras^{V12}-expressing hepatocytes, and crizotinib inhibits phopho-c-Met signaling in the liver

C57BL/6 mice were fed ND or HFD for three months and subjected to hydrodynamic tail vein injection with plasmid encoding GFP-N-Ras^{V12}-IRES-luciferase. Some of the ND and HFD-fed mice were orally administered with 50 mg/kg crizotinib thrice. After six days of HTVi, isolated livers from mice were subjected to immunofluorescence. E-cadherin (**a**), Vimentin (**b**) and phospho-c-Met (**c**) (red) and GFP (**a–c**) (green) were detected in liver section. The cell nuclei were stained using DAPI (blue). Scale bars, 40 μm.

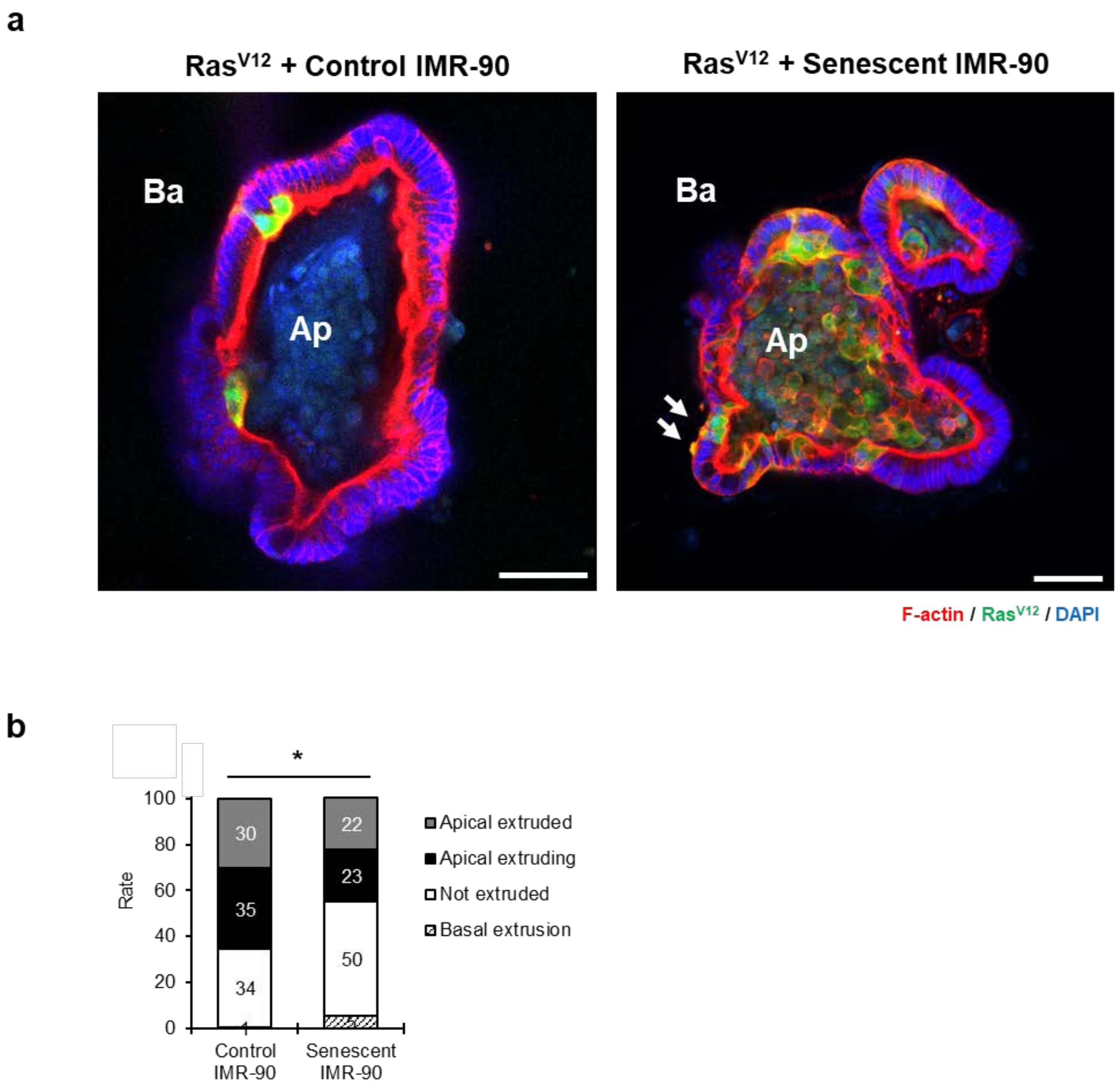

Figure S10. Coculture with senescent fibroblasts inhibits the apical extrusion and promotes basal extrusion of Ras^{V12}-transformed cells *ex vivo*

a, Immunofluorescence images of intestinal organoids of cell competition model mice (villin-Cre-*ERT2*, *LSL-Ras^{V12}-IRES-eGFP*) after treatment with 100 nM tamoxifen coculturing with nonsenescent IMR90 cells (control) or Ras^{V12}-induced senescent IMR90 cells. “Extruding”: with their nucleus apically shifted, but still attached to the basement membrane. “Apical extruded”: completely detached from the basement membrane and translocated into the apical lumen. “Basal extrusion”: completely translocated under the epithelial cell layer and invading the basal membrane. The white arrowhead indicates basally extruded Ras^{V12}-GFP cells. Ba and Ap stand for the basal and apical sides, respectively. Scale bars, 50 μm. **b**. Quantification of the apically extruded, extruding, not extruded, or basally extruding MDCK-pTR GFP-Ras^{V12} cells *ex vivo*. Data are presented as mean ± standard error of the mean. **P* < 0.05 for extruded + extruding Ras^{V12}-GFP cells coculturing with control or senescent IMR-90 cells by unpaired two-tailed t-test; three independent experiments.

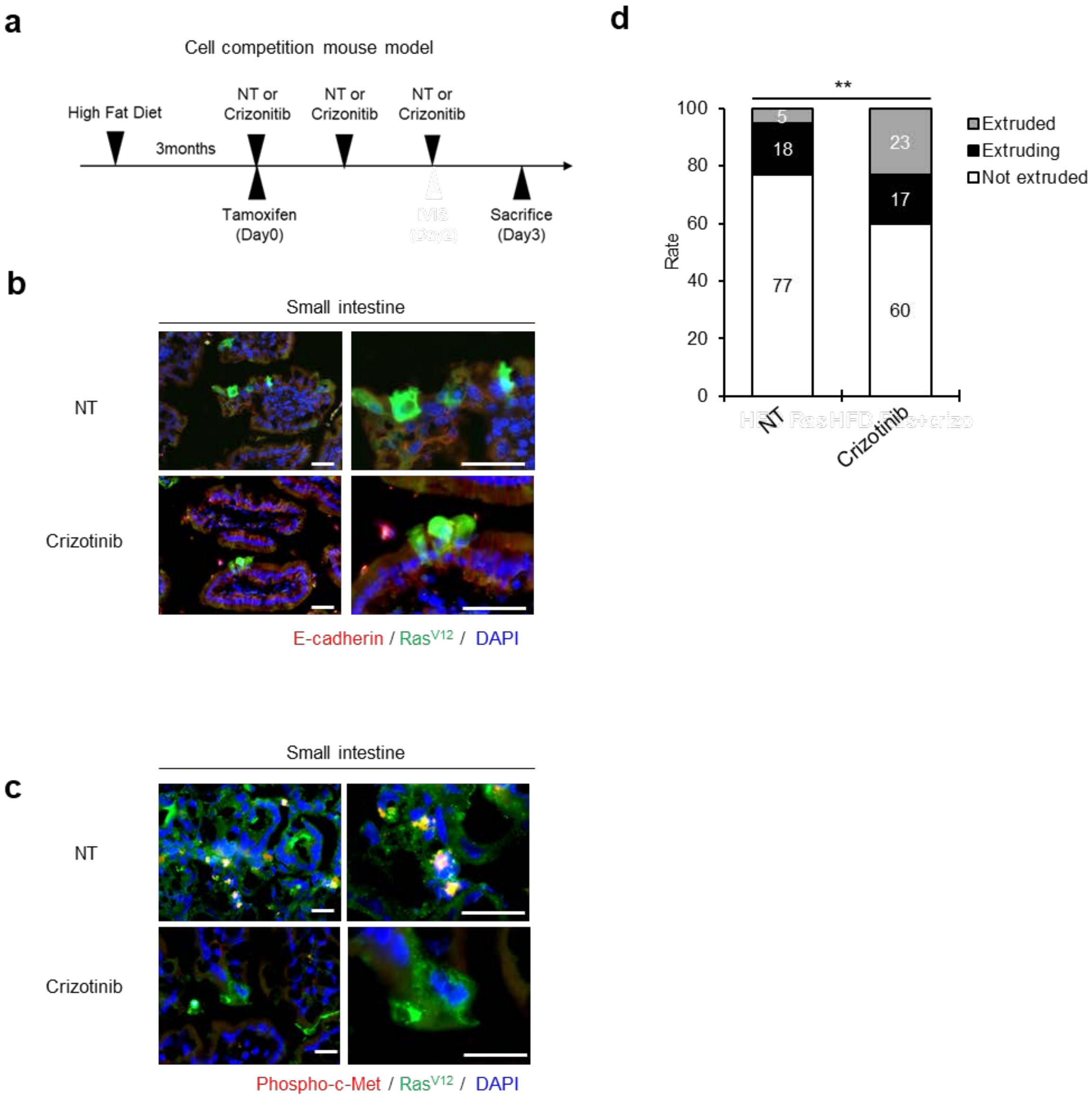

Figure S11. Crizotinib promotes apical extrusion of the Ras^{V12}-transformed cells in the small intestine of HFD-fed cell competition mice

a, Timeline of the experimental procedure. Cell competition model mice (villin-Cre-*ERT2*, *LSL-Ras^{V12}-IRES-eGFP*) were fed HFD for 3 months and orally administered 50 mg/kg crizotinib. **b**, **c**, Immunofluorescence images (**b**, **c**) and quantification analysis (**d**) of Ras^{V12} cells in the epithelium of the small intestine. E-cadherin (red) (**b**), phospho-c-Met (red) (**c**), and Ras^{V12} signals (green) were detected in small intestine section, and DNA was stained using DAPI (blue). Scale bars, 20 μm. p = 0.001. **P < 0.01 by chi-square test.
