# Supplementary material for: Hepatocyte growth factor derived from senescent cells attenuates cell competition-induced apical elimination of oncogenic cells
Source: Nat Commun. 2022 Jul 18;13:4157. doi: 10.1038/s41467-022-31642-4 (PMC9293948; doi:10.1038/s41467-022-31642-4)
Supplement: Supplementary file 3 — Reporting Summary [file 41467_2022_31642_MOESM3_ESM.pdf]

nature portfolio

Corresponding author(s): Akiko Takahashi

Last updated by author(s): Jun 20, 2022

Reporting Summary

Nature Portfolio wishes to improve the reproducibility of the work that we publish. This form provides structure for consistency and transparency in reporting. For further information on Nature Portfolio policies, see our Editorial Policies and the Editorial Policy Checklist.

Statistics

For all statistical analyses, confirm that the following items are present in the figure legend, table legend, main text, or Methods section.

n/a	Confirmed	
☐	☒	The exact sample size (*n*) for each experimental group/condition, given as a discrete number and unit of measurement
☐	☒	A statement on whether measurements were taken from distinct samples or whether the same sample was measured repeatedly
☐	☒	The statistical test(s) used AND whether they are one- or two-sided<br>*Only common tests should be described solely by name; describe more complex techniques in the Methods section.*
☒	☐	A description of all covariates tested
☐	☒	A description of any assumptions or corrections, such as tests of normality and adjustment for multiple comparisons
☐	☒	A full description of the statistical parameters including central tendency (e.g. means) or other basic estimates (e.g. regression coefficient) AND variation (e.g. standard deviation) or associated estimates of uncertainty (e.g. confidence intervals)
☐	☒	For null hypothesis testing, the test statistic (e.g. *F*, *t*, *r*) with confidence intervals, effect sizes, degrees of freedom and *P* value noted<br>*Give P values as exact values whenever suitable.*
☒	☐	For Bayesian analysis, information on the choice of priors and Markov chain Monte Carlo settings
☒	☐	For hierarchical and complex designs, identification of the appropriate level for tests and full reporting of outcomes
☒	☐	Estimates of effect sizes (e.g. Cohen's *d*, Pearson's *r*), indicating how they were calculated

Our web collection on statistics for biologists contains articles on many of the points above.

Software and code

Policy information about availability of computer code

Data collection	Microscopic data: LSM710 confocal microscope (Zeiss)<br>Quantitative real-time PCR data: StepOnePlus PCR system (Applied Biosystems)<br>Immunoblotting data: FUSION imaging system (Vilber-Lourmat)<br>ELISA and cytokine assay: FUSION imaging system (Vilber-Lourmat)<br>Histological data: Immunofluorescence microscope (Carl Zeiss AG)<br>Bioluminescent imaging data: IVIS Imaging System (PerkinElmer)<br>Microarray analysis: Agilent Feature Extraction v11.0.1.1.
Data analysis	GO analysis: DAVID(the database for annotation, visualization and integrated discovery, version 6.8)<br>Immunoblotting analysis: NIH Image J software (version 1.57)<br>Bioluminescent imaging analysis: Living Image Software (PerkinElmer)<br>ELISA and cytokine analysis: NIH Image J software<br>Statistical analysis: R software for statistical computing (64-bit version 3.6.1)

For manuscripts utilizing custom algorithms or software that are central to the research but not yet described in published literature, software must be made available to editors and reviewers. We strongly encourage code deposition in a community repository (e.g. GitHub). See the Nature Portfolio guidelines for submitting code & software for further information.

Data

Policy information about availability of data

All manuscripts must include a data availability statement. This statement should provide the following information, where applicable:

- Accession codes, unique identifiers, or web links for publicly available datasets
- A description of any restrictions on data availability
- For clinical datasets or third party data, please ensure that the statement adheres to our policy

The datasets generated during this current study are available in a publicly accessible repository.

Field-specific reporting

Please select the one below that is the best fit for your research. If you are not sure, read the appropriate sections before making your selection.

☒ Life sciences ☐ Behavioural & social sciences ☐ Ecological, evolutionary & environmental sciences

For a reference copy of the document with all sections, see nature.com/documents/nr-reporting-summary-flat.pdf

Life sciences study design

All studies must disclose on these points even when the disclosure is negative.

Sample size	The sample sizes used in this study were determined based on common practice of the described experiments and the need to have sufficient statistical power.
Data exclusions	No data were excluded.
Replication	All experiments were performed at least 2 times. For some experiments, n=3 independent biological samples were used to perform statistical calculations. All data were successfully replicated and came similar outcomes.
Randomization	For animal studies, mice were randomly allocated to experimental groups.
Blinding	All data presented in this study did not require the use of blinding.

Reporting for specific materials, systems and methods

We require information from authors about some types of materials, experimental systems and methods used in many studies. Here, indicate whether each material, system or method listed is relevant to your study. If you are not sure if a list item applies to your research, read the appropriate section before selecting a response.

Materials & experimental systems

n/a	Involved in the study	
☐	☒	Antibodies
☐	☒	Eukaryotic cell lines
☒	☐	Palaeontology and archaeology
☐	☒	Animals and other organisms
☒	☐	Human research participants
☒	☐	Clinical data
☒	☐	Dual use research of concern

Methods

n/a	Involved in the study	
☒	☐	ChIP-seq
☒	☐	Flow cytometry
☒	☐	MRI-based neuroimaging

Antibodies

Antibodies used	Ras (Oncogene Research Products, #OP41), lamin-B1 (Abcam, #ab16048), GAPDH (Proteintech, #60004-1-lg), Phospho-Met (Tyr1234/1235) (D26) XP Rabbit mAb (Cell Signaling Technology, #3077), ECL anti-mouse IgG horse raddish peroxidase-linked whole antibody (GE Healthcare, #NA931V),ECL anti-rabbit IgG horse raddish peroxidase-linked whole antibody (GE Healthcare, #NA934V), a-SMA (Sigma, #A5228), Desmin (Sigma, #D1033), HGF (R&D Systems, #AF-294-NA), CXCL10 (R&D, #AF-466-NA), 53BP1 (Santa cruz, #sc-22760) , E-cadherin Monoclonal Antibody (ECCD-2) (Invitrogen, #131900), Vimentin (Abcam,#ab92547), NRAS (Proteintech, #10724-1-AP), GFP (Abcam, #ab13970), γ-H2AX (Millipore, #05-636), phosphor-Ser/Thr ATM/ATR (pST/Q) substrate (Cell Signaling Technology, #2851), Cellular Senescence Detection Kit - SPiDER-βGal (Dojindo, #SG03), Recombinant Anti-Ki67 antibody [SP6] (abcam, #ab16667), Alexa Fluor 568 phalloidin (Thermo Fisher Scientific, #A12380), Alexa Fluor 488 goat anti-mouse (Thermo Fisher Scientific, #A11001), 594 donkey anti-goat antibodies (Thermo Fisher Scientific, #A11058), Alexa Fluor 594 goat anti-Rabbit IgG (H+L) Cross-Adsorbed Secondary Antibody (Thermo Fisher Scientific, A11012) and Alexa Fluor 488 goat Anti-Chicken IgY H&L (Abcam,

	#ab150169).
Validation	All antibodies used in this study were commercially available antibodies and were validated by providers and other researchers. The data sheets are available from the website as follows:<br>Ras, https://www.merckmillipore.com/products/antibody/OP41<br>lamin-B1, https://www.abcam.co.jp/lamin-b1-antibody-nuclear-envelope-marker-ab16048.html<br>GAPDH, https://www.ptglab.com/products/GAPDH-Antibody-60004-1-Ig.htm<br>Phospho-Met (Tyr1234/1235) , https://www.cellsignal.co.uk/products/primary-antibodies/phospho-met-tyr1234-1235-d26-xp-rabbit-mab/3077<br>ECL anti-mouse IgG, horseradish peroxidase-linked whole antibody (GE Healthcare, #NA931V), https://www.cytivalifesciences.co.jp/catalog/0428.html<br>ECL anti-rabbit IgG, horseradish peroxidase-linked whole antibody (GE Healthcare, #NA934V), https://www.cytivalifesciences.co.jp/catalog/0428.html<br>a-SMA, https://www.sigmaaldrich.com/JP/ja/product/sigma/a5228<br>Desmin, https://www.sigmaaldrich.com/JP/ja/product/sigma/d1033?<br>CXCL10, https://www.rndsystems.com/products/mouse-cxcl10-ip-10-crg-2-antibody_af-466-na?utm_source=citeab&utm_medium=referral&utm_campaign=product&utm_term=primaryantibodies<br>53BP1, https://www.scbt.com/p/53bp1-antibody-h-300?productCanUrl=53bp1-antibody-h-300&_requestid=278943<br>Phalloidin, https://www.thermofisher.com/order/catalog/product/A12381<br>Vimentin, https://www.abcam.co.jp/vimentin-antibody-epr3776-cytoskeleton-marker-ab92547.html<br>HGF, https://www.rndsystems.com/products/human-hgf-antibody_af-294-na<br>NRAS, https://www.thermofisher.com/antibody/product/NRAS-Antibody-Polyclonal/10724-1-AP<br>GFP, https://www.abcam.co.jp/gfp-antibody-ab13970.html<br>γ-H2AX, https://www.merckmillipore.com/JP/ja/product/Anti-phospho-Histone-H2A.X-Ser139-Antibody-clone-JBW301,MM_NF-05-636<br>ATM/ATR (pST/Q), https://www.cellsignal.jp/products/primary-antibodies/phospho-ser-thr-atm-atr-substrate-antibody/2851<br>SPiDER-βGal, https://www.dojindo.co.jp/products/SG03/<br>Recombinant Anti-Ki67 antibody, https://www.abcam.co.jp/ki67-antibody-sp6-ab16667.html<br>Alexa Fluor 568 phalloidin, https://www.thermofisher.com/order/catalog/product/A12380#/A12380<br>E-cadherin Monoclonal Antibody (ECCD-2), https://www.thermofisher.com/antibody/product/E-cadherin-Antibody-clone-ECCD-2-Monoclonal/13-1900<br>Alexa Fluor 488 goat anti-mouse, https://www.thermofisher.com/antibody/product/Goat-anti-Mouse-IgG-H-L-Highly-Cross-Adsorbed-Secondary-Antibody-Polyclonal/A32723<br>Alexa Fluor 594 goat anti-rabbit, https://www.thermofisher.com/antibody/product/Goat-anti-Rabbit-IgG-H-L-Highly-Cross-Adsorbed-Secondary-Antibody-Polyclonal/A32740<br>Alexa Fluor 594 donkey anti-goat, https://www.thermofisher.com/antibody/product/Donkey-anti-Goat-IgG-H-L-Cross-Adsorbed-Secondary-Antibody-Polyclonal/A-11058<br>Alexa Fluor 594 goat anti-Rabbit IgG (H+L) Cross-Adsorbed Secondary Antibody, https://www.thermofisher.com/antibody/product/Goat-anti-Rabbit-IgG-H-L-Cross-Adsorbed-Secondary-Antibody-Polyclonal/A-11012<br>Alexa Fluor 488 goat Anti-Chicken IgY H&L, https://www.thermofisher.com/antibody/product/Goat-anti-Chicken-IgY-H-L-Secondary-Antibody-Polyclonal/A-11039

Eukaryotic cell lines

Policy information about cell lines

Cell line source(s)	TIG-3 cells were obtained from the Japanese Cancer Research Resources Bank. IMR-90 cells were obtained from American Type Culture Collection. Human hepatic stellate cells were purchased from DS Pharma Biomedical. Mouse primary hepatic stellate cells were isolated from mouse liver of C57BL/6J wild type mouse. MDCK cells were kindly provided by Dr. W. Birchmeier.
Authentication	Authentication of mouse hepatic stellate cells was not necessary because they were developed in our lab. Other cells were obtained from bioresource bank and Companies. Therefore, authentication was not performed by ourselves.
Mycoplasma contamination	All cell lines were negative for mycoplasma contamination.
Commonly misidentified lines (See ICLAC register)	No commonly misidentified cell lines were used in this study.

Animals and other organisms

Policy information about studies involving animals; ARRIVE guidelines recommended for reporting animal research

Laboratory animals	C57BL/6J wild type male mouse of 7 to 8 weeks old were used.
Wild animals	No wild animals involved in this study.
Field-collected samples	This study did not involve samples collected from the field.
Ethics oversight	All animal experimental procedures were conducted using protocols approved by the JFCR Animal Care and Use Committee, according to the relevant guidelines and regulations.

Note that full information on the approval of the study protocol must also be provided in the manuscript.
